# Supplementary material for: From sequence to enzyme mechanism using multi-label machine learning
Source: BMC Bioinformatics. 2014 May 19;15:150. doi: 10.1186/1471-2105-15-150 (PMC4229970; doi:10.1186/1471-2105-15-150)
Supplement: Additional file 2 — Java code of ml2db. Additional file ml2db_code.tar.gz contains the Java source code to run the multi-label machine learning experiments and save the results to database. The code’s Javadoc is included. [file 1471-2105-15-150-S2.zip › additional file 2/ml2db/utils/doc/uk/ac/standrews/utils/main/guiutils/SimpleRadioButtonPanel.html]

SimpleRadioButtonPanel


JavaScript is disabled on your browser.


- Overview
- Package
- Class
- Use
- Tree
- Deprecated
- Index
- Help

- Prev Class
- Next Class

- Frames
- No Frames

- All Classes

- Summary:
- Nested |
- Field |
- Constr |
- Method

- Detail:
- Field |
- Constr |
- Method


uk.ac.standrews.utils.main.guiutils

## Class SimpleRadioButtonPanel

- java.lang.Object
- - uk.ac.standrews.utils.main.guiutils.SimpleRadioButtonPanel

- ---

    

  ```
  public class SimpleRadioButtonPanel
  extends java.lang.Object
  ```

- - ### Field Summary

    Fields

    | Modifier and Type | Field and Description |
    | --- | --- |
    | `java.lang.Object` | `choiceEvent` |
  - ### Constructor Summary

    Constructors

    Constructor and Description

    `SimpleRadioButtonPanel(java.lang.String[] radioOptions,
    java.lang.String[] comments)`

    Generates a simple radio button popup
  - ### Method Summary

    Methods

    | Modifier and Type | Method and Description |
    | --- | --- |
    | `java.lang.String` | `getChosenOption()` |
    | `static void` | `main(java.lang.String[] args)` For testing |
    | `void` | `setChosenOption(java.lang.String option)` |

    - ### Methods inherited from class java.lang.Object

      `equals, getClass, hashCode, notify, notifyAll, toString, wait, wait, wait`

- - ### Field Detail


    - #### choiceEvent

      ```
      public java.lang.Object choiceEvent
      ```
  - ### Constructor Detail


    - #### SimpleRadioButtonPanel

      ```
      public SimpleRadioButtonPanel(java.lang.String[] radioOptions,
                            java.lang.String[] comments)
      ```

      Generates a simple radio button popup

      Parameters:
      :   `radioOptions` - the options for each radio button
      :   `comments` - the text/comments beside each radio button
  - ### Method Detail


    - #### main

      ```
      public static void main(java.lang.String[] args)
      ```

      For testing

      Parameters:
      :   `args` -


    - #### getChosenOption

      ```
      public java.lang.String getChosenOption()
      ```


    - #### setChosenOption

      ```
      public void setChosenOption(java.lang.String option)
      ```


- Overview
- Package
- Class
- Use
- Tree
- Deprecated
- Index
- Help

- Prev Class
- Next Class

- Frames
- No Frames

- All Classes

- Summary:
- Nested |
- Field |
- Constr |
- Method

- Detail:
- Field |
- Constr |
- Method
